# Barriers and Facilitators to HIV Testing Among Zambian Female Sex Workers in Three Transit Hubs

**DOI:** 10.1089/apc.2017.0016

**Published:** 2017-07-01

**Authors:** Michael M. Chanda, Amaya G. Perez-Brumer, Katrina F. Ortblad, Magdalene Mwale, Steven Chongo, Nyambe Kamungoma, Catherine Kanchele, Andrew Fullem, Leah Barresi, Till Bärnighausen, Catherine E. Oldenburg

**Affiliations:** ^1^ John Snow, Inc., Lusaka, Zambia.; ^2^ Department of Sociomedical Sciences, Columbia University Mailman School of Public Health, New York, New York.; ^3^ Department of Global Health and Population, Harvard T.H. Chan School of Public Health, Boston, Massachusetts.; ^4^ John Snow, Inc., Boston, Massachusetts.; ^5^ Department of Epidemiology, Harvard T.H. Chan School of Public Health, Boston, Massachusetts.; ^6^ Faculty of Medicine, Institute of Public Health, University of Heidelberg, Heidelberg, Germany.; ^7^ Africa Health Research Institute, Mtubatuba, South Africa.; ^8^ Francis I. Proctor Foundation, University of California, San Francisco, California.

**Keywords:** HIV testing, female sex workers, Zambia

## Abstract

Zambia has a generalized HIV epidemic, and HIV is concentrated along transit routes. Female sex workers (FSWs) are disproportionately affected by the epidemic. HIV testing is the crucial first step for engagement in HIV care and HIV prevention activities. However, to date little work has been done with FSWs in Zambia, and little is known about barriers and facilitators to HIV testing in this population. FSW peer educators were recruited through existing sex worker organizations for participation in a trial related to HIV testing among FSWs. We conducted five focus groups with FSW peer educators (*N* = 40) in three transit towns in Zambia (Livingstone, Chirundu, and Kapiri Mposhi) to elicit community norms related to HIV testing. Emerging themes demonstrated barriers and facilitators to HIV testing occurring at multiple levels, including individual, social network, and structural. Stigma and discrimination, including healthcare provider stigma, were a particularly salient barrier. Improving knowledge, social support, and acknowledgment of FSWs and women's role in society emerged as facilitators to testing. Interventions to improve HIV testing among FSWs in Zambia will need to address barriers and facilitators at multiple levels to be maximally effective.

## Introduction

Female sex workers (FSWs) are disproportionately affected by the HIV epidemic globally.^1^ In Sub-Saharan Africa, FSWs have 12.4 times the odds of HIV infection compared with general population women of reproductive age.^1^ Testing for HIV is a critical first step in the HIV care cascade for addressing the HIV epidemic, by allowing for timely linkage to care to initiate treatment and reduce viral load, and prevent onward transmission to sexual partners. However, major gaps in the HIV care cascade remain for FSWs.^2^ Recent population-based evidence from Zimbabwe demonstrated that only 64% of FSWs were aware of their HIV positive status.^2^ Similarly, knowledge of status has been reported to be low in other regions of Sub-Saharan Africa among FSWs.^3,4^ Interventions to improve HIV testing coverage for FSWs are warranted, the development of such interventions must include an understanding of existing barriers and facilitators to HIV testing from the perspective of this vulnerable community.

FSWs face multilevel barriers to accessing healthcare services, which can limit HIV testing coverage. Diverse barriers to accessing HIV testing have been noted, such as criminalization of sex work, healthcare provider stigma, distance or time required to go to a clinic, and fear of testing.^5–8^ For FSWs in particular, stigma may be a particularly salient barrier.^6,9,10^ FSWs may experience both HIV-related and sex work-related stigma operating in tandem and/or cumulatively.^10–12^ Stigma has been broadly defined as negative attitudes, relative powerlessness, and loss of status related to membership in a particular group or characteristic. Stigma can be experienced as enacted (explicit actions against an individual), perceived (such as the expectation that enacted stigma will occur), or internalized or self-stigma (the internalization of enacted and perceived stigma that results in negative attitudes about the self).^13^ Conversely, factors such as social support and accessibility have been discussed as enabling access to HIV testing.^7,8^ Given the complex and intersecting nature of factors influencing HIV testing, such barriers and facilitators are highly contextual: interventions that work in one setting may be unlikely to be effective in others.

Zambia has a generalized HIV epidemic, with HIV prevalence of ∼15% in the general population.^14^ A considerable amount of HIV-related stigma remains in Zambia, with a high proportion of people living with HIV reporting social exclusion, gossip, and harassment because of their HIV status.^15^ Stigma remains an important barrier to HIV testing in the general population as well as key populations,^16,17^ and trust in healthcare workers has shown to be a facilitator to voluntary counseling and testing (VCT).^17^ The Zambian HIV epidemic is heterogeneous, with the highest prevalence found in transit areas and along rail lines. Correspondingly, HIV prevalence is thought to be higher among Zambian FSWs than among the general population, but limited evidence exists.^18^ A 2009 survey among FSWs conducted in four transit towns found that 21% of FSWs reported never testing for HIV. Primary reasons for not testing for HIV were reported to be anticipated fear related to testing and not wanting to know status.^19^ Sex work is effectively illegal in Zambia, which can limit access to HIV testing and other preventative services. Further, the lack of sex worker organizations currently operating in Zambia means that there is little advocacy for development of competent care for this population.

Given the unique HIV prevention needs of FSWs in Zambia, we conducted focus groups with FSW peer educators to elicit community norms surrounding HIV testing among FSWs in transit towns (including border towns and trucking hubs). The aim of this formative research was to gain a more in-depth understanding of perceived barriers and facilitators of HIV testing among FSWs.

## Methods

In August 2016, semistructured focus groups (*N* = 5, total participants = 40) were conducted with peer educators who either currently identified as FSWs or had previously participated in sex work. Written informed consent was collected from each participant. Participants received 50 kwacha (∼US$5) as compensation for their time participating in this study. The Institutional Review Boards at the Harvard T.H. Chan School of Public Health in Boston, United States and ERES Converge in Lusaka, Zambia, approved the study.

### Study setting

This study took place in three transit hubs in Zambia—Livingstone, Chirundu, and Kapiri Mposhi. Livingstone and Chirundu are situated on the Zambia–Zimbabwe border, and are busy cross-border stops for trucks traveling throughout southern Africa. Livingstone, home to Victoria Falls, is also a major tourist destination in Zambia. Kapiri Mposhi is on a major transit route for truckers traveling north to the Copperbelt Region, the Democratic Republic of the Congo, and Tanzania. There is a weigh station in Kapiri Mposhi at which all truck drivers are required to stop, which can result in delays of multiple days in the town. The sex work industry in each of the three study sites is driven primarily by transit and the trucking industry, although there is also a sex tourism industry in Livingstone. Sex workers in these areas are highly mobile, frequently moving between towns, for example, traveling to the Copperbelt around payday for mining workers.

### Participants and procedures

Peer educators were recruited for participation in a randomized controlled trial at each of the three study sites.^20^ Peer educators were recruited through contacts from two former sex worker projects operating in Zambia, Corridors of Hope and Tasinta. Corridors of Hope was a President's Emergency Plan for AIDS Relief (PEPFAR)-funded project implemented by FHI360 that provided HIV prevention services, including HIV testing and counseling services, to individuals living in border and transportation communities who were members of higher risk groups, such as sex workers and truck drivers. The project concluded in September 2014. Tasinta offered HIV prevention services and opportunities to leave sex work for women in Zambia. Tasinta ceased operations in 2015. The discontinuation of these programs led to reduced options for HIV testing for FSWs in these settings. Peer educators were recruited by study staff and selected for participation in the study based on criteria including their willingness to participate in the full duration of the main study (∼6 months) and their reliability. All peer educators were at least 18 years of age, were cisgender women (i.e., assigned female sex at birth and currently identify as a woman), and self-reported being current or former sex workers.

A random sample of 16 peer educators per study site (8 per focus group) was selected for inclusion in focus group discussions before peer educator training or any other study activities. Focus group discussions were designed to elicit a baseline understanding of the availability of HIV testing, current practices, barriers, and facilitators to HIV testing, as well as peer educators' views on HIV self-testing. Focus group semistructured questions and probes focused specifically around (1) HIV testing practices, (2) barriers and facilitators to HIV testing, (3) HIV self-testing perceptions, and (4) the influence of knowing HIV status on sexual behaviors.

### Guiding theoretical framework and analytic strategy

Focus group discussions were employed to obtain an understanding of community-level perspectives^21^ about barriers and facilitators to HIV testing among FSWs by community leaders currently or previously engaged in sex work. Interested in how the complex associations between multilevel factors, including individual, social, and structural, influenced (both as a barrier and facilitator) HIV testing, we were theoretically guided by a modified social ecological model. This theoretical framework has been previously used to highlight HIV vulnerabilities among key populations across diverse sociocultural contexts.^22^

All focus group discussions were facilitated in Nyanja, Bemba, and/or English by native-speaking facilitators, audio recorded, and transcribed verbatim. To elicit community-level expertise from the key informants, focus group probes specifically aimed to promote participant–participant interactions and an understanding of social discourses to detect collective and shared experiences. Guided by immersion crystallization, A.P.B. and C.E.O. analyzed the transcripts based on an inductive and deductive approach to identify themes and relationships between themes. Iterative drafts of the code book, differences in coding application, and understanding of emergent themes were discussed among all authors. Qualitative analysis was conducted using Dedoose Version 5.0.11, (2014, Los Angeles, CA: SocioCultural Research Consultants, LLC, www.dedoose.com).

## Results

Across five focus groups in three distinct Zambian transit hubs, discussions supported emerging themes related to barriers and facilitators to HIV testing at three inter-related levels: individual (intrapersonal), community/close social network (meso-level), and structural.

### Barriers to HIV testing

Participants discussed a range of barriers that might prevent FSWs from engaging in routine HIV testing. At the individual level, permutations of narratives describing fear of discrimination and stigma were frequently discussed. Key informants importantly highlighted the intersection between internalized stigma and fear regarding an HIV diagnosis and sex work.

1B. *Others do have self-stigma, because she knows that she's a sex worker she says I can't go there, when I go there I will just test positive. (Livingstone FG#2)*

2B. *Others also fail to accept the results. Somebody decides to go and test but she is not sure or ready. It makes them to start imagining what could happen when tested positive. Before you have an HIV test, you need to be ready to accept the result. (Livingstone FG#1)*

Further, descriptions of internalized stigma were heightened by anticipated fears regarding lack of confidentiality and concerns that if tested, others in their community would find out about their status.

3B. *Most of the people are shy going to the hospital (Kapiri FG#3)*

4B. *It is difficult to test because some think if I get tested this same person testing me, will tell others “that's the one I tested she is sick of HIV.” (Kapiri FG#3)*

Lack of information was also described as a potential barrier and interwoven with notions of individual versus community-level responsibilities of knowing ones' status.

5B. *A lot of people are ignorant, like my sister has said, some know that there's HIV and AIDS but they don't want to test, to others its lack of information, they don't know anything about HIV and AIDS. (Livingstone FG#2)*

Beyond the perception of anticipated stigma and discrimination, participants highlighted other barriers directly linked to interactions with community members and/or individuals within their social network. Previously mentioned fears related to confidentiality at the intrapersonal level linked to concerns at the meso-level that there would be gossip if others learned their status.

6B. *Seeing her before she even enters the VCT room, they will say there she is, maybe she's already sick that's why she's come to test. (Livingstone FG#2)*

7B. *They might be afraid that if I go for the test and my friend knows that I am positive there will be discrimination or my friend will begin to tell people that I am positive. (Chirundu FG#4)*

8B. *In the case of the clinic, there is congestion and Sex Workers feel shy. They need privacy so that they are not seen by certain people. (Livingstone FG#1)*

Narratives additionally underscored potential meso-level barriers including intimate partner violence and HIV stigma and sex worker stigma as enacted by healthcare providers.

9B. *Even when you go to test as a couple. You get tested. One will be found positive and the other negative. Now violence will start. Fighting will start. Maybe the marriage will end there where you went to test. (Chirundu FG#4)*

The intersection between HIV stigma and sex work stigma highlighted the potential consequence of defining sex workers as a population at increased vulnerability to HIV, noting that HIV tests may be forced and not consented to by sex workers (Quote 10B).

10B. *Because what usually happens in clinics nowadays is that even when you only suffer from headache, they will make sure you undergo an HIV screening whether you like it or not. They stopped asking if you would like to test for HIV. (Livingstone FG#1)*

Further, discussions explicitly highlighted this intersection by giving examples of “judgmental attitude(s)” by providers.

11B. *Even counselors, as they go around providing HIV counseling and testing services would be making stigmatizing remarks in the presence of the client, for instance as they put on gloves they would be saying “lest I get infected,” Therefore such remarks may eventually attract a reaction from a client's side such as chasing the health care provider due to judgmental attitude about the client's possible HIV status. (Livingstone FG#1)*

These recurrent themes of HIV stigma and sex worker stigma were further described as a structural level barrier, not only deterring individual behavior based on attitudes and beliefs of the self and toward providers but additionally of institutions as a whole (e.g., clinics and hospitals).

12B. *The sex workers also go to the hospital but they are not many who accept to go the hospital. Like here in Chirundu most are afraid to go to the hospital. They are shy. They have stigma (Chirundu FG#4)*

Key informants provide contextual knowledge as to how space in hospitals is organized, noting that “queuing up for antiretrovirals (ARVs)” is publicly visible and can identify individuals as living with HIV.

13B. *Some it's the fear of queuing up for ARVs once they test HIV positive. So they would rather not test. (Livingstone FG#1)*

14B. *And the other thing that scares people. Because like at our hospital there, they can test you like when you are pregnant. They catch you with this disease. Just there and then when you are seated with your friends. You will be picked. And one can notice that something has happened with our friend. You'll be taken to their office to do their test. Right there and then they start you on medication. And when going to get the medicine it is the same block they are using, antenatal, child health, under five and what else, family planning, TB. It is the same block open like this (points to the meeting space/open space). They even see that these on this line are going to get the medicine. And it is not everyone who is open. So you find a person feels that the health is not ok that they should test but they are afraid of the same thing. (Chirundu FG#4)*

15B.*At our Urban Clinic it's very difficult to collect drugs because the office where you go and collect drugs from is just by the way and everyone knows that those who collect drugs from here are HIV positive. (Kapiri FG#3)*

Sex work as an occupation was also described as a structural barrier because of the nocturnal hours of the occupation that lead some sex workers to be employed during routine hours of operation and/or financial loss incurred because of taking time to seek an HIV test rather than pursing employment opportunities.

16B. *Most of the times as you said it is difficult for sex workers to go at the hospital go for HIV testing… they are always busy doing the work they do. (Kapiri FG#3)*

17B. *It's difficult to them because they feel that once they move away from their homes and go to the clinic, they will waste time, maybe a man will come to have sex with other and give her money; so she will feel like when she goes to the clinic she will waste time instead of waiting to make money that will come (Livingstone FG#2)*

### Facilitators for HIV testing

All five focus groups also discussed strategies across the individual level (intrapersonal), community/close social network (meso-level), and structural level to promote HIV testing among FSWs. At the individual level (Quotes 1F–6F), empowerment because of knowledge and occupational status as a sex worker was described as key to promoting women to test for HIV.

1F. *She's a sex worker, she knows the kind of job she's doing…it's important to go for a test so that each person knows their status. (Livingstone FG#2)*

2F. *There are times that I as a sex worker had no information, and if any person with information comes to sensitize me on issues of HIV and benefits of testing, I may be motivated to go and take an HIV test. (Livingstone FG#1)*

Pregnancy was frequently described as a facilitator to engaging in HIV testing. Narratives described a spectrum of rationales related to pregnancy from enacted agency to protect one's child, family planning and seeking birth control, and HIV testing being a routine aspect of prenatal screenings.

3F. *In my view, it is not common among sex workers to test for HIV. Because just as my sister mention earlier that sex workers know the kind of work they are involved into, it's not common for them to test. Unless she is pregnant or she gets sick and goes to the hospital, they will be able to test her for HIV, and then she will know her HIV status. (Livingstone FG#1)*

4F. *Sometimes it is pregnancy, if you have care for your baby you want to protect it, definitely you will go for VCT. (Livingstone FG#2)*

5F. *These sexual workers do not want to be pregnant or having children. Hence, most of the times they test when they are getting contraceptives, this is the only time they have to test because they have no free time. (Kapiri FG#3)*

6F. *They go [to get an HIV test] when they are pregnant. There when pregnant they find it. (Chirundu FG#4)*

Participants suggested that strategies that facilitated HIV testing were also closely linked to community and social network support. Meso-level facilitators described included sexual partners (Quotes 7F–9F), peer network (i.e., fellow FSWs and friends) (Quotes 10F–11F), experiences with family living with or dying from HIV (Quote 12F), and nonjudgmental experiences with healthcare provides (Quote 13F).

7F. *Others go to get tested when sometimes they find a boyfriend. The he asks you, have you ever been tested? Then you answer him that no I have not. Sometimes he wants to know your status before you get into a friendship. He will tell you that let us go and get tested. So sometimes that is when you can go. You and get tested so that you know how both of you are. Then when you find out there that is when you can go ahead with the programme. (Chirundu FG#4)*

8F. *I know it is difficult to convince a man to use a condom but you just have to know how, tell him that both of you do not know your status to protect each other you are to use a condom, if he insists then tell him that you are to first go and know each other's status. (Kapiri FG#3)*

9F. *I think sometimes it could be that you have a client who refuses to use a condom during sexual intercourse. He may say, if I don't use a condom, you will be given more money, let us say K1000 but with a condom it's K500. But you go for a K1000 and after you go home, you realize you did not do the right thing, so you will think of going for an HIV test. (Livingstone FG#1)*

10F. *Just as the other speaker mentioned that a sex worker will get along with a fellow sex worker. She becomes free and is able to share anything she feels like sharing. (Livingstone FG#1)*

11F. *Sometimes where we live, as friends someone may be complaining and you observe some symptoms, sometimes we help and encourage such friends taking them, going with them we get tested together. (Kapiri FG#3)*

12F. *What is causing people now to test for HIV, they have seen a lot of people in families were dying because not going to the Hospitals to test or test from the providers in the community, they have seen a lot of orphan that have been left because of this same disease, even though they didn't want to test, they will remember a sister who died because of the same disease and left us these orphans; so many are no longer refusing. (Livingstone FG#2)*

13F. *There must be a good relation because most of the time let's say the Hospital is near and the doctor knows that you are sexual worker he/she must not be unconcerned or biased because of your status but must care testing you and giving you results. (Kapiri FG#3)*

Notably, experiences with family living with or dying from HIV overlapped with descriptions of orphaned children (Quote 12F), reinforcing intrapersonal themes, suggesting the importance of pregnancy and family planning in facilitating HIV testing.

Structural environments, primarily contexts related to gender norms (Quote 14F), and antistigma practices, including a theatrical skit (Quote 15F) and peer education (Quote 16F), were discussed by participants as empowering FSWs to increase general knowledge related to HIV testing.

14F. *What makes women test for HIV…is we are also important in society, we have dependents, some of us are bread winners. Hence, the need to get tested for HIV. (Kapiri FG#3)*

15F. *When people go to sensitize like at a market place and may also perform a skit just like the way Mosi-o-tunya group does it. After performing a skit, they showcase some dance and later teach about HIV on how test is done, immediately people, who will have understood the messages, will soon start accessing the service. (Livingstone FG#1)*

16F. *Like these organizations that have come which incorporate sex workers; that sex worker will call her friends and recruits them for a group discussion and then they are given knowledge and education, many of them end up accessing testing. (Livingstone FG#2)*

Recognition of the importance of women in society was highlighted as an empowering cultural norm (Quote 14F) and paralleled descriptions of enacted agency at the individual level (Quote 1F) that was linked to descriptions of sex work as an occupation. Notably, norm-changing interventions, such as public performance (Quote 15F) and institutions that practice antidiscrimination policies (e.g., hiring of sex workers), were described as important facilitators to increase education and promote HIV testing.

## Discussion

Narratives from community-based peer educators who were active or previously engaged in female sex work highlighted multilevel barriers and facilitators to HIV testing among FSWs in Zambia. Across focus groups, the intersecting levels (i.e., individual, community, and structural) underscored stigma and discrimination as a pervasive barrier to HIV testing. Previous work has shown the importance of stigma as a barrier to HIV testing and linkage to care among FSWs in diverse settings.^9,23–25^ Stigma-mitigating interventions may, therefore, be helpful for improving access to HIV testing in this population, and a multipronged approach may be required to address stigma-related barriers at multiple levels. Such interventions will need to be developed and implemented in a way that responds to the specific needs of the population. A previous study of a skills-building and collectivization intervention, addressing individual and structural levels of sex work-related stigma for FSWs, in India demonstrated mixed effects, with some groups reporting empowerment and some reporting reluctance to self-identify as sex workers.^26^ However, the majority of stigma-related interventions focus only on individual-level stigma and only a single dimension of stigma for key populations (e.g., HIV-related stigma or sex work-related stigma).^27^ For FSWs in Zambia, dissemination of knowledge to (individual-level) and among/between FSWs (community-level) regarding the importance of testing may help to dispel fears. Structural interventions focusing on clinic and hospital settings to promote safe and confidential spaces may also improve HIV testing for FSWs. In addition to safer spaces, interventions aimed at decreasing healthcare provider stigma toward FSWs from clinicians will be important to optimize HIV testing in this highly stigmatized population.

Peer educators similarly identified several key facilitators across multiple levels. Perhaps the most important facilitator was the description of community and societal support that spanned both meso- and structural levels.^28,29^ These results highlight the importance of social networks. Specifically, sexual partners and peer networks help to empower FSWs to bridge existing barriers and test for HIV. Our findings also indicated the importance of contextualizing these facilitators within a broader structural context. Of note, narratives highlighted experiences with family living, or with dying from HIV and leaving behind orphaned children. The lived reality of Zambia, with a generalized HIV epidemic and in which participants noted they see community members dying of HIV-related causes, emerged as an important motivator for testing for HIV among FSWs. Further, participants acknowledged economic responsibility (i.e., they were breadwinners and had children depending on them), resulting in increased motivation to test routinely for HIV. Acknowledging the lived realities of FSWs, rather than simply focusing on work lives, may result in more comprehensive, acceptable, and sustainable HIV testing interventions.

An important facilitator for HIV testing that emerged in all focus groups was pregnancy. HIV testing during pregnancy is a major component of prevention of mother-to-child transmission programs. In generalized epidemic settings such as Zambia, pregnant women are recommended to test for HIV every 3 months during their pregnancy, although in practice there is a substantial gap in repeat HIV testing coverage among pregnant women in Zambia.^30^ However, among FSWs, pregnancy intentions and reproductive health needs are often overlooked.^31,32^ Our results indicate that multiple facets of pregnancy and childbearing, including enacted agency to protect one's child, family planning and seeking birth control, and routine testing as part of antenatal care services, are important motivators for seeking HIV testing. For FSWs, antenatal care and family planning services will likely be important venues for HIV testing, and stigma mitigating interventions for FSWs should consider focusing on these care delivery points.

These results should be interpreted in the context of several limitations. Data are derived from a limited sample size (*N* = 5 focus groups) of 40 peer educator key informants across three distinct transit towns in Zambia. Thus, these narratives provide a snapshot into the key barriers and facilitators for HIV testing among FSWs, but findings are not generalizable to all FSWs in Zambia or other settings. Focus groups were designed to elicit community-level norms related to HIV testing. Although we anticipate this would minimize social desirability bias, there may be other important themes arising from individual interviews designed to elicit individual-level behaviors. Finally, additional qualitative scholarship is also needed on positive interactions and experiences with healthcare providers to provide insight into existing strategies to combat sex worker-based stigma and discrimination and promote FSW-specific cultural competence within healthcare facilities in Zambia.

These results document the complex and multilevel barriers and facilitators to HIV testing among FSWs in three transit towns in Zambia. HIV is concentrated within Zambia along transit routes, and understanding barriers and facilitators nested within this context may lead to better interventions to improve HIV testing coverage. Themes related to stigma and discrimination at multiple levels, including healthcare provider stigma and fear of sex worker disclosure, indicate that stigma-reducing interventions may be necessary to improve HIV testing. Ultimately, interventions to improve HIV testing for FSWs in Zambian transit towns will need to consider community-level expertise from FSWs and incorporate a multilevel framework to achieve maximal results.
